# A naturalistic paradigm simulating gaze-based social interactions for the investigation of social agency

**DOI:** 10.3758/s13428-019-01299-x

**Published:** 2019-11-11

**Authors:** Marie-Luise Brandi, Daniela Kaifel, Juha M. Lahnakoski, Leonhard Schilbach

**Affiliations:** Independent Max Planck Research Group for Social Neuroscience, Max Planck Institute of Psychiatry, Munich, Germany

**Keywords:** Agency, Social interaction, Gaze simulation, Eyetracking

## Abstract

*Sense of agency* describes the experience of being the cause of one’s own actions and the resulting effects. In a social interaction, one’s actions may also have a perceivable effect on the actions of others. In this article, we refer to the experience of being responsible for the behavior of others as *social agency*, which has important implications for the success or failure of social interactions. Gaze-contingent eyetracking paradigms provide a useful tool to analyze social agency in an experimentally controlled manner, but the current methods are lacking in terms of their ecological validity. We applied this technique in a novel task using video stimuli of real gaze behavior to simulate a gaze-based social interaction. This enabled us to create the impression of a live interaction with another person while being able to manipulate the gaze contingency and congruency shown by the simulated interaction partner in a continuous manner. Behavioral data demonstrated that participants believed they were interacting with a real person and that systematic changes in the responsiveness of the simulated partner modulated the experience of social agency. More specifically, gaze contingency (temporal relatedness) and gaze congruency (gaze direction relative to the participant’s gaze) influenced the explicit sense of being responsible for the behavior of the other. In general, our study introduces a new naturalistic task to simulate gaze-based social interactions and demonstrates that it is suitable to studying the explicit experience of social agency.

*Sense of agency* refers to feeling oneself to be responsible for reactions in the environment, and it is believed to depend on an implicit coupling of one’s own action and the perceived outcome. Sense of agency is thought to be one major aspect of the *minimal self*, the basic and pre-reflective sense of having a self that can interact with the world around us (Gallagher, 2000). In a mechanistic model of agency, Synofzik, Vosgerau, and Voss (2013) emphasized that the feeling of being in control is based on an implicit feeling as well as an explicit judgment of experienced agency.

Although many studies have focused on the sense of agency during motor actions (e.g., Chambon, Sidarus, & Haggard, 2014), less is known about this phenomenon in social interactions. Consistent with hypothesized models that ascribe the experience of agency to the ability to link the action to the corresponding outcomes (Blakemore, Wolpert, & Frith, 2000), a similar connection can be expected for social responses of another person after initiating a social action (Friston & Frith, 2015; Kunde, Weller, & Pfister, 2018; Wolpert, Doya, & Kawato, 2003). In this article, we will refer to the concept of agency within a social interaction as social agency (Brandi, Kaifel, Bolis, & Schilbach, 2019; Recht & Grynszpan, 2019). Social agency therefore describes the feeling of being responsible for a reaction of another person. Due to the importance of social contact, humans are highly sensitive to such social feedback and the responsiveness of others to one’s own actions (Carlin & Calder, 2013; Schilbach et al., 2013; Schilbach et al., 2010; Stoyanova, Ewbank, & Calder, 2010). We learn such action-outcome associations early during development and the experiences made during social interactions are likely to influence the development of the self as well as future social encounters (Aitken & Trevarthen, 1997; Fonagy, Gergely, & Target, 2007; Neisser, 1991; Trevarthen & Aitken, 2001). However, the characteristics and the underlying mechanisms of social agency are still ambiguous. Previous research provides important evidence that both an implicit and explicit sense of agency is present within a social context (Pfister, Obhi, Rieger, & Wenke, 2014; Stephenson, Edwards, Howard, & Bayliss, 2018; Weiss, Herwig, & Schütz-Bosbach, 2011) and that gaze behavior provides a useful aspect to study agency both in general (Gregori Grgič, Crespi, & de’Sperati, 2016) as well as in social situations (Pfeiffer et al., 2012, 2014; Pfeiffer, Timmermans, Bente, Vogeley, & Schilbach, 2011; Stephenson et al., 2018). In a recent publication, Stephenson et al. applied the widely used intentional binding paradigm including social cues. The intentional binding effect is based on the concept that humans experience a shorter timeframe between self-generated actions and their outcomes compared with two events that were not caused by themselves. In the mentioned study it was shown that after initiating a gaze shift, gaze following (indicated by a picture of a face) could elicit an implicit sense of agency as well as an explicitly reported experience of control and therefore provide important evidence for the existence of social agency.

Since agency is strongly dependent on the associations of actions and their outcomes, it seems clear that the evaluation of these processes within the social domain should preferably be studied in a realistic interactive social situation. Gaze-contingent eyetracking paradigms represent a promising method to simulate social interactions (Redcay, Kleiner, & Saxe, 2012; Schilbach et al., 2010; Wilms et al., 2010) and study the experience of social agency. This method uses a computer algorithm, which presents social stimuli according to the measured gaze behavior of the participant, creating an interactive simulation in real-time. A great advantage of this method is that the responses of the interaction partner can be controlled and experimentally manipulated. Two important parameters that can be manipulated are the congruency and the contingency of the observed interaction partner. Congruent observed behavior can be characterized as being similar to one’s own behavior or as being in line with one’s expectations. As an example, gaze following, as compared to gaze aversion, can be described as congruent behavior since the gaze of oneself and the interaction partner is similarly directed to the same goal. Contingency describes the dynamics and the temporal relatedness of the observed behavior toward one’s own behavior. Manipulating gaze parameters characterizing the behavior of a simulated interaction partner may be used to modulate the perceived responsiveness and as a result the elicited experience of agency (Pfeiffer et al., 2012; Recht & Grynszpan, 2019). These parameters include the time the interaction partner needs to respond to one’s own behavior and the type of reaction such as gaze following as well as direct gaze.

So far, most studies using gaze-contingent paradigms for studying social interaction present computer-generated anthropomorphic characters as interaction partners (Frädrich, Nunnari, Staudte, & Heloir, 2018; Redcay et al., 2012; Schilbach et al., 2010; Wilms et al., 2010). Often cover stories are introduced including a pseudo-interaction partner to be present, in order to make participants believe they are interacting with a real person (e.g., Schilbach et al., 2010). The use of such anthropomorphic characters is very useful to study feedback processes constitutive of social interaction while providing a high level of experimental control (Frädrich et al., 2018; Redcay et al., 2012; Schilbach et al., 2010; Wilms et al., 2010). On the other hand, a digital representation of a human being might not be as ecologically valid as a natural image of a real person. One core element of social interactions is not only experiencing someone responding to oneself, but also attributing a mind as well as intentionality to the interaction partner and therefore sensing that the other person experiences agency themselves (Brandi et al., 2019; Caruana, de Lissa, & McArthur, 2017a; Caruana, Spirou, & Brock, 2017c; Crivelli & Balconi, 2010, 2015). Research has shown that the level of mind attribution to an interaction partner changes gaze behavior within the interaction (Abubshait & Wiese, 2017; Wiese, Wykowska, Zwickel, & Müller, 2012; Wykowska, Wiese, Prosser, & Müller, 2014). Furthermore, it has been demonstrated that the attribution of mind is affected by the humanlike appearance of the interaction partner. In particular, studies on human-robot interaction suggest that interaction partners with realistic human features are more likely to be attributed with an own mind and agency (Abubshait & Wiese, 2017; Martini, Gonzalez, & Wiese, 2016). We therefore believe that increasing the humanlike appearance of an interaction partner (e.g., by using real dynamic images of a person) also influences the experience of social agency. The mentioned research further emphasizes the importance of realistic interaction partners, as compared to computer-generated characters in experiments studying social interaction. The attribution of agency toward an interaction partner might also influence one’s own experience of agency within an interaction. It is therefore necessary to study the sense of agency in a realistic interactive setting and to improve experimental procedures by using naturally responsive facial stimuli to simulate a realistic social interaction.

The present study had two main goals: The first was to evaluate a newly developed experimental paradigm comprising a video-based gaze-contingent simulation of a social interaction and, second, to measure the explicit experience of social agency within an interaction by manipulating the responsiveness of the simulated social interaction partner. Essential for the evaluation of the paradigm was the experience of the participants within the simulation. We hypothesized that the experimental procedure presented here simulates a gaze-based social interaction and that participants believe they are interacting with a real person. A cover story was used to make participants believe they are in an online social interaction with another person in order to study gaze behavior during video-based digital communication. As part of the evaluation of the newly developed experimental procedure, we also investigated different experimental settings. In a believable simulation, it should not be necessary for an interaction partner to be present at the experiment to convey the experience of a real online-interaction. On the basis of experience from previous studies in which we used similar cover stories successfully (e.g. Richardson et al., 2012; Schilbach et al., 2010), we predicted that the reported experiences in the interaction would be similar in two tested experimental procedures: In one, the interaction partner shown in the simulation was present in the room with the participant; in the other, the simulated interaction partner was not present for the measurement. Furthermore, we hypothesized that the explicit sense of social agency would be dependent on the experimentally manipulated level of responsiveness shown by the simulated interaction partner. The simulated behavior followed the same programmed experimental time course for all participants, with phases of altering levels of responsiveness. High responsiveness is characterized by contingent as well as congruent gaze behavior, whereas low responsiveness is characterized by a lack of congruency and/or contingency of behavior. It was expected that the participants interacting with the simulation would be able to sense these changes in responsiveness and therefore to experience different levels of social agency accordingly.

## Method

### Participants

Fifty-two healthy individuals participated in the study. The group sizes were chosen on the basis of prior studies that had included between 21 and 30 participants for their experiments (Pfeiffer et al., 2012; Pfeiffer et al., 2011). No formal power calculation was performed to estimate the samples size. The study and all procedures were approved by the local ethics committee and informed consent was obtained from the participants. All participants had normal or corrected-to-normal vision and no history of psychiatric or neurological disorders. Since it can be assumed that high autistic traits might influence the measures of interest, the participants filled out the Autism Spectrum Quotient questionnaire (AQ; Baron-Cohen, Wheelwright, Skinner, Martin, & Clubley, 2001). No participant reached the cutoff of 32 (Baron-Cohen et al., 2001) that is suggestive of autism spectrum disorder. Two participants were excluded (one participant withdrew consent; the other participant was familiar with similar experimental procedures and suspicious about the cover story already before participating in the experiment). As a result, 50 participants entered the first analysis validating the experimental procedure (*M*_age_ = 24.24, *SD*_age_ = 4.63; 29 female; *M*_AQ_ = 16.04, *SD*_AQ_ = 5.19). To test two different experimental procedures, the study population was divided into two groups. Both group A (*M*_ageA_ = 23.40, *SD*_ageA_ = 3.45; 15 female; *M*_AQA_ = 15.60, *SD*_AQA_ = 4.88) and group B (*M*_ageB_ = 25.08, *SD*_ageB_ = 5.52; 14 female; *M*_AQB_ = 16.48, *SD*_AQB_ = 5.55) included 25 participants each. The groups did not differ significantly in age [*t*(48) = – 1.291, *p* = *.*203, *d* = – 0.365], or AQ score [*t*(48) = – 0.596, *p* = *.*554, *d* = – 0.168]. Six participants were excluded from the analysis evaluating perceived agency, due to technical problems in the data collection of button presses during the task. Therefore, 21 participants in group A (*M*_ageA_ = 23.52, *SD*_ageA_ = 3.53; 12 female; *M*_AQA_ = 15.47, *SD*_AQA_ = 4.86) and 23 in group B (*M*_ageB_ = 25.13, *SD*_ageB_ = 5.70; 12 female; *M*_AQB_ = 16.73, *SD*_AQB_ = 5.69) were included in the analysis of perceived agency. Age [*t*(42) = – 1.110, *p* = *.*273, *d* = 0.338] and AQ [*t*(42) = – 0.787, *p* = *.*436, *d* = 0.239] did not differ significantly between these subgroups.

### Stimuli

**Fig. 1:**
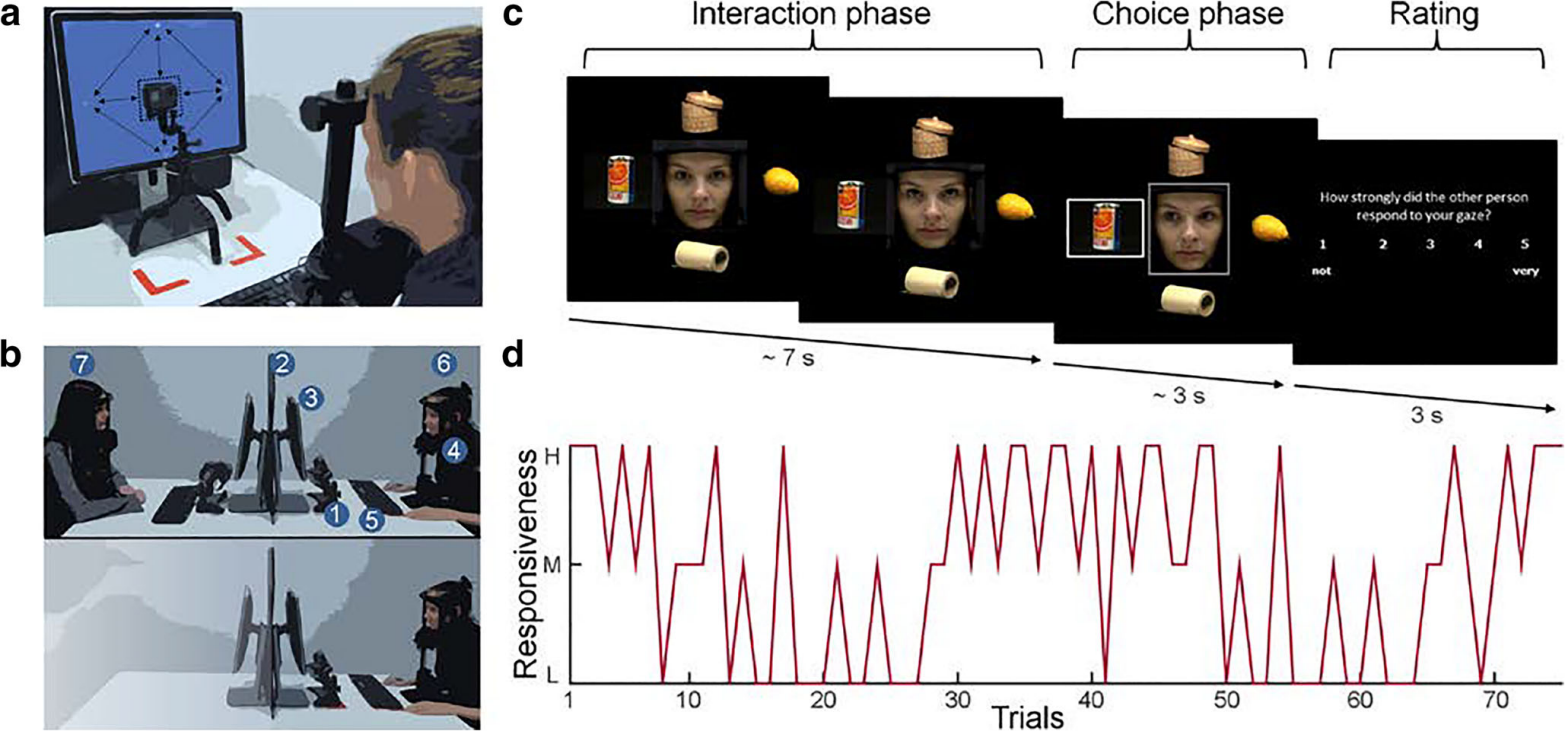
Experimental setup and design. (A) For generation of the video stimuli, the recorded person was seated in front of a monitor with a camera in front of the middle of the screen, with the head positioned in a headrest. The recorded person has to switch between five different gaze positions, indicated by a fixation cross (up, left, right, and down) or a box around the camera (center), leading to 20 different saccades. (B) Illustration of the experimental setup for both experimental groups. (Upper panel) Experimental setup for group A: 1, eyetracker; 2, black partition screen; 3, monitor; 4, headrest; 5, keyboard; 6, participant; 7, pseudo-interaction partner shown in the simulation. (Lower panel) Setup for group B, with no pseudo-interaction partner present. (C) During each trial, the participant interacts with an interaction partner (shown in the video), whose gaze behavior is controlled by the computer. Each interaction trial starts with a free interaction phase. In the choice phase, both the participant and the simulated interaction partner choose one of four objects. After this, during the rating phase, the participant has to judge the responsiveness of the simulated interaction partner on a 5-point scale. (D) Time course of the responsiveness (H, high; M, medium; L, low) shown by the simulated interaction partner over the 75 trials of the whole experiment.

In the present, newly developed paradigm, video recordings of different gaze behaviors of a real human interaction partner were implemented as stimuli simulating a realistic gaze-based interaction. To create such stimuli, the recorded interaction partner (recruited from the research group) was seated in front of a computer screen with their head on a headrest and was instructed to show a relaxed, neutral face. A camera (Hero 5 Black, GoPro Inc., San Mateo, CA, USA) was placed at the center of the screen to record the face of the interaction partner. A script programmed in Presentation software (Version 18.0, Neurobehavioral Systems, Inc., Berkeley, CA, www.neurobs.com) was used to direct the gaze of the interaction partner according to a predefined schedule to five different gaze positions, as depicted in Fig. 1A, as well as a short sequence with closed eyes. The latter sequence was not used for the experimental procedures in this study. The sequence of gaze shifts were recorded 15 times, to create a stimulus set of 300 video sequences of 20 different gaze shifts between all pairs of targets. The gaze shift sequences ended with the instruction for the interaction partner to close their eyes, which was terminated by a beep from the controller computer. The beep signaling the end of each round was subsequently used to cut the 15 sets of gaze shifts to separate videos, to avoid cumulative timing errors toward the end of the recording session. The videos were then cut into short video clips, each showing a single, full gaze shift from a starting position to an ending position, based on the gaze schedule used during the recording. At the ending position, the gaze rested for 3–4 s. The schedule of starting points of the video clips was manually adjusted by a constant time shift until the saccades were aligned within a few tens of milliseconds from the start of each video, to account for individual differences in reaction times. Finally, the videos were examined visually to ensure their quality. Of particular importance was that there were no strong changes in head motion and facial expression between the different sequences, which could influence the believability of the simulation by including unrealistic jumps during the continuous display. Videos with strong movements were therefore excluded. In addition to the videos, several objects selected from the Amsterdam Library of Object Images were used as stimuli in the experiment (Geusebroek, Burghouts, & Smeulders, 2005).

### Experimental setup

The experimental setup consisted of a monitor (Intel Core i5-4690 CPU 3.5GHz, 8GB RAM, MS Windows 7 Enterprise, refresh rate = 59Hz) and an EyeLink 1000+ (SR Research, Ottawa, ON, Canada) eye-tracker positioned in front of the monitor. The participants head was rested on a headrest during the experiment. The distance of the eyes of the participants and the monitor was circa 670 mm and for the camera of the eyetracker 500 mm. The experiment was presented with Presentation software (Version 18.0, Neurobehavioral Systems, Inc., Berkeley, CA, www.neurobs.com) with a resolution of 1,024 × 768 pixels. A standard computer keyboard was used to record participants’ responses. A second, identical setup was positioned opposite to the participant. The setups were separated by a black screen (Fig. 1B).

### Experimental paradigm

The present paradigm was based on an established method, which uses eyetracking to present gaze-contingent stimuli (Pfeiffer, Vogeley, & Schilbach, 2013; Schilbach et al., 2010; Wilms et al., 2010). During the experimental procedure, the position of the participant’s gaze was recorded with an eyetracker and entered into an algorithm that controlled the presentation of the experimental stimuli according to the position of the participant’s gaze. The video clips of gaze shifts were presented without gaps, creating the illusion of continuous gaze behavior and a real-time social interaction.^1^ The implemented algorithm can be modified in order to systematically manipulate different parameters of the simulated interaction partner’s gaze behavior in order to manipulate the sense of social agency of the human interactor. The gaze of the interaction partner was systematically varied on a trial-by-trial basis by controlling the probability of contingency and congruency of the simulated gaze. Here, the following factors were varied: (1) *direct gaze* (does the simulated interaction partner return eye contact or not), (2) *reaction latency* (how fast/slow does the simulated interaction partner react), and (3) *gaze following* (does the simulated interaction partner follow the gaze toward a third point of attention or not). Decreasing the frequency of gaze-following as well as direct gaze (decreased congruency) and increasing the reaction latency on a trial-by-trial basis (decreased contingency) was expected to decrease the responsiveness of the interaction partner and therefore the intensity of the sense of social agency. In the present experiment, we included three levels of responsiveness: *High*, *medium*, and *low*. In trials showing a *highly* responsive interaction partner, the simulation was programmed to always return eye contact and to follow the gaze of the participant in 100% of cases (high congruency). Additionally, the reaction latency was programmed to be jittered around 400 ms (between 350 and 450 ms), which is a reaction time known to elicit the feeling of relatedness and contingency (Pfeiffer et al., 2012). In trials in which the simulated interaction partner showed a *medium* level of responsiveness, the simulation was programmed to return eye contact and follow the gaze in 50% of cases, while the reaction latency was longer (between 750 and 850 ms). During trials showing a *low* responsiveness, the simulation did not react to the participant’s gaze at all, but followed a randomly chosen gaze path with jittered latencies between the gaze shifts, independent of the participant’s gaze. The three levels were equally represented within the experiment (25 trials each, leading to 75 trials). The time course of the whole experiment, depicting the changing levels of responsiveness, is shown in Fig. 1D. The schedule was the same for all participants.

As is depicted in Fig. 1C, each trial started by showing the face of the simulated interaction partner as well as pictures of four different objects. During the *interaction* phase, the participants’ were told to interact freely and explore the objects for 7 s together with the simulated interaction partner. After this phase, a gray box was shown around the face of the simulated interaction partner, which indicated the start of the so-called *choice* phase. In this phase, the participants were instructed to select one of the four objects by looking at it. After selection, the chosen object was marked by a gray box. The simulated interaction was programmed to react with the same level of responsiveness in the choice phase as in the interaction phase. The choice phase was primarily included to motivate the participants to explore the objects together with the simulated interaction partner. Each interaction trial ended with a rating in which participants had to evaluate how strongly they had the impression that the interaction partner had reacted toward their own gaze, on a five-point-scale (German: “Wie stark hat die andere Person auf Ihren Blick reagiert?”; English translation: “How strongly did the other person respond to your gaze?”). The scale ranged from 1 = *not at all* to 5 = *very*. The participants were instructed to evaluate the responsiveness according to their experience considering the whole trial. The rating measured, on a trial-by-trial basis, whether participants noticed differences in the action–outcome association of the performed and perceived gaze behavior. The measure therefore reflects an explicit measure of changing levels of experienced social agency.

### Procedure

To enhance the ecological validity of the experimental setup, a cover story was used to make the participants believe that they were really interacting with another person via a video-connection. Participants were told that the purpose of the study was to analyze gaze behavior during digital communication, since nowadays, an increasing amount of communication is done via digital video-chats (e.g. Skype). In one group (group A), the interaction partner in the video was introduced to the participant as a naïve second participant. During the experiment the pseudo-interaction partner sat opposite to the participant at one of two computers with the same experimental setup as the participant (Fig. 1B). The two setups were separated by a large black partition screen so the participants could not see each other directly and both participants were given earplugs to discourage additional communication that could have influenced the believability of the simulation. A comparable cover story has been successfully used in several similar studies using animated avatars as indirect representations of an interaction partner (Caruana, McArthur, Woolgar, & Brock, 2017b; Pfeiffer et al., 2014; Schilbach et al., 2010; Wilms et al., 2010). In group B, no pseudo-interaction partner was present in the experiment. We explained to the participants that the interaction partner was sitting in a different room with another experimenter. Before the experiment started, a phone call was simulated, in which the two experimenters aimed to coordinate the simultaneous start of the experiment. Except for the presence of the interaction partner, the experimental procedure was the same for both groups. The interacting pairs were always gender-matched. The experimental session started after participants had given their consent. They were asked to fill out a short questionnaire on their experience with digital communication. Afterward, participants were introduced to the paradigm and then started the experiment. The experiment lasted approximately 20 min, and after finishing, the participants filled out a second set of questionnaires, as well as the AQ. A detailed description of the items in the questionnaires can be found in the next section. The questionnaires were acquired in a digital form on the experimental computer. After the first set of questions, a text was presented describing the simulation and cover story, to debrief the participants, which was followed by further questions. Participants were informed that they could withdraw their consent after having learned about the deception.

### Questionnaires

Participants were asked to fill out several questionnaires before and after the experiment. The pre-experiment questionnaire included demographic questions and items on their personal experience in digital communication. The latter questions were only used for the cover story and were not relevant for the present experimental results and will therefore not be discussed further.

To evaluate the plausibility of the simulation and compare the participants’ experiences of the simulated interaction between the two experimental groups, a self-composed questionnaire was used, which comprised items from two established questionnaires on the concept of presence in virtual environments (Witmer & Singer, 1998) and social presence in telecommunication (Nowak, 2001; Nowak & Biocca, 2003). The general concept of *presence* describes the experience of being in an environment or situation, even though a person is not physically in this situation (Witmer & Singer, 1998). Three items were included from the Presence Questionnaire (PQ) particularly measuring the factor of feeling in control (CF; Witmer & Singer, 1998) of the events in the simulation. Social presence on the other hand is a measure used in telecommunication and describes the experience of being in a social or communicative situation in which a person has access to another person’s mind while using an interface for interaction (Nowak, 2001; Nowak & Biocca, 2003). Fourteen items were taken from the Social Presence Questionnaire (SPQ; Nowak, 2001; Nowak & Biocca, 2003). Three items covered the concept of telepresence (TP; the experience of being within an environment), five items the concept of self-reported co-presence (SRP; the own feeling of connectedness with a partner within an interaction) and another five items the perceived co-presence (PC; the perceived connectedness of the partner with yourself within an interaction). An additional item from our questionnaire measured the experience of social presence (SP; Item 17: “I had the impression to interact with a ‘real’ person.”), which was of particular interest for measuring whether the participants had the feeling of being in a real online interaction. The phrasing of some items was slightly adjusted in order to fit the experimental situation of this study, while the meaning was mainly retained. All items were rated on a five-point Likert scale. Table 1 lists all items of interest in their original form, the rephrased form, the used German translation, the corresponding original questionnaire, and the measured concept. To get an average score for each of the presented concepts of CF, TP, SRP, and PC, negative items (9, 11, 14, and 15) were reversed and the median score of each concept was calculated. Scores above the neutral value of 3 indicated a stronger accordance with the specific concept of experienced presence, whereas scores below 3 suggested no or reduced experience of presence.

**Table 1:** Questionnaire items

No.	Original	Rephrased	German	Source	Concept
1	How much were you able to control events?		*Zu welchem Grad konnten Sie die Ereignisse kontrollieren?*	PQ	CF
2	How responsive was the environment to actions that you initiated (or performed)?	How responsive was your interaction partner to actions that you initiated (or performed)?	*Wie responsiv war Ihr Interaktionspartner gegenüber Aktionen, die Sie initiierten* (*oder durchführten)?*	PQ	CF
3	Were you able to anticipate what would happen next in response to the actions that you performed?	To what extend were you able to anticipate what would happen next in response to the actions that you performed?	*Zu welchem Grad konnten Sie vorhersagen was als Reaktion auf die von Ihnen durchgeführten Handlungen passiert?*	PQ	CF
4	How involving was the experience?		*Wie involvierend war die Erfahrung?*	SP	TP
5	How intense was the experience?		*Wie intensiv war die Erfahrung?*	SP	TP
6	To what extent did you feel immersed in the environment you saw/heard?		*Zu welchem Grad tauchten Sie in die Situation ein?*	SP	TP
7	I was interested in talking to my interaction partner.	I was interested in interacting with my partner.	*Ich hatte Interesse daran mit meinem Partner zu interagieren.*	SP	SRP
8	I was intensely involved in this interaction.		*Ich beteiligte mich stark an der Interaktion.*	SP	SRP
9	I wanted to maintain a sense of distance between us.		*Ich wollte ein Gefühl der Distanz zwischen uns aufrecht erhalten.*	SP	SRP
10	I tried to create a sense of closeness between us.		*Ich versuchte ein Gefühl der Nähe zwischen uns herzustellen.*	SP	SRP
11	I did not want a deeper relationship with my interaction partner.		*Ich wollte die Beziehung zu meinem Interaktionspartner nicht vertiefen.*	SP	SRP
12	My interaction partner was interested in talking to me.		*Mein Interaktionspartner hatte Interesse daran mit mir zu interagieren.*	SP	PC
13	My interaction partner was intensely involved in our interaction.		*Mein Interaktionspartner beteiligte sich stark an unserer Interaktion.*	SP	PC
14	My interaction partner created a sense of distance between us.		*Mein Interaktionspartner stellte ein Gefühl von Distanz zwischen uns her.*	SP	PC
15	My interaction partner seemed detached during our interaction.		*Mein Interaktionspartner schien während unserer Interaktion unbeteiligt zu sein.*	SP	PC
16	My interaction partner created a sense of closeness between us.		*Mein Interaktionspartner stellte ein Gefühl von Nähe zwischen uns her.*	SP	PC
17	To what extent did your partner seem “real”?	I had the impression to interact with a real person.	*Ich hatte den Eindruck mit einer „realen“ Person zu interagieren.*	SP	SP

Items 1–6 used a five-point scale, from 1 = *not at all* to 5 = *very*; for Items 7–17, the scales were 1 = *completely disagree*, 2 = *mainly disagree*, 3 = *partly agree*, 4 = *mainly agree*, 5 = *completely agree*.

In addition to this questionnaire, participants were asked to indicate in an open commentary field what they noticed about the interaction: “About the interaction I noticed, that ___.” After the debriefing (presented in the form of a written description) participants were asked to indicate whether they were aware of interacting with a simulation and further to indicate in a drop-down menu at which time point of the experiment they might have suspected this (e.g., during the experiment or explicitly at which item of the questionnaire).

### Data analysis: Plausibility and experience of simulation

Data analysis of the button-presses and questionnaires, along with the statistical analysis, was performed in MATLAB (R2015a, The MathWorks, Inc., Natick, MA, USA). Nonparametric tests were used since the acquired data were ordinal. To evaluate the plausibility of the simulation, a so-called plausibility criterion was defined that comprised two critical items from the previously described questionnaire. One item included the open commentary field collected before the debriefing. It was evaluated to be critical if participants expressed disbelief about the simulation or cover story in the commentary field. Second, if participants indicated after debriefing that they were aware of the simulation during the experiment, the participant was considered critical of the cover story. If both of the two items were uncritical (meaning that neither of the two items indicated disbelief) a score of zero was given. Otherwise, the participant received a score of one, indicating that the participant did not believe they were interacting online with a real person. A Fisher’s exact test was used to test whether the two groups differed with respect to the plausibility-criterion. Furthermore, it was analyzed if Item 17 (“I had the impression to interact with a ‘real’ person.”) was significantly different from the neutral score of 3 (*partly agree*) for the whole group with a Wilcoxon signed-rank test. Assuming that participants had the impression of interacting with a real person, a tendency toward the score of 5 (*completely agree*) was expected. For each of the four concepts of presence, also, a Wilcoxon signed-rank test was used to test against the neutral score of 3. Higher scores indicated a higher experience of presence, as compared to scores below 3. Differences in Item 17 and the experienced presence in the simulation for the concepts TP, SRP, CF, and PC between the two groups were tested with a Mann–Whitney *U* test. For the descriptive statistics of these measures, the median and interquartile range (IQR) were calculated and reported.

### Data analysis: Experience of social agency

To evaluate whether participants’ experience of social agency changed as a function of varying levels of responsiveness of the interaction partner, the median of the trial-by-trial ratings from all participants for each trial was calculated and correlated to the experimentally modeled time course of the responsiveness levels by using a Spearman’s rank correlation. A Mann–Whitney *U* test was used to test whether the median rating of the experienced intensity differed between the two groups of participants.

## Results

### Believability ratings

Ninety-six percent of the participants (48 out of 50) believed that they were interacting with a real person, as indicated by a score of 0 for the plausibility criterion. Two participants in experimental group B indicated after debriefing that they had been aware of the simulation during the experiment. One of the two participants also reported in the commentary field that they were aware that the person shown in the experiment was pre-recorded. However, the presence of the interaction partner did not lead to a significant difference in the frequency of the plausibility criterion between the groups (zero out of the 25 members of group A and two out of the 25 members of group B showed a plausibility criterion of 1; *p* = *.*489, Fisher’s exact test)

**Fig. 2:**
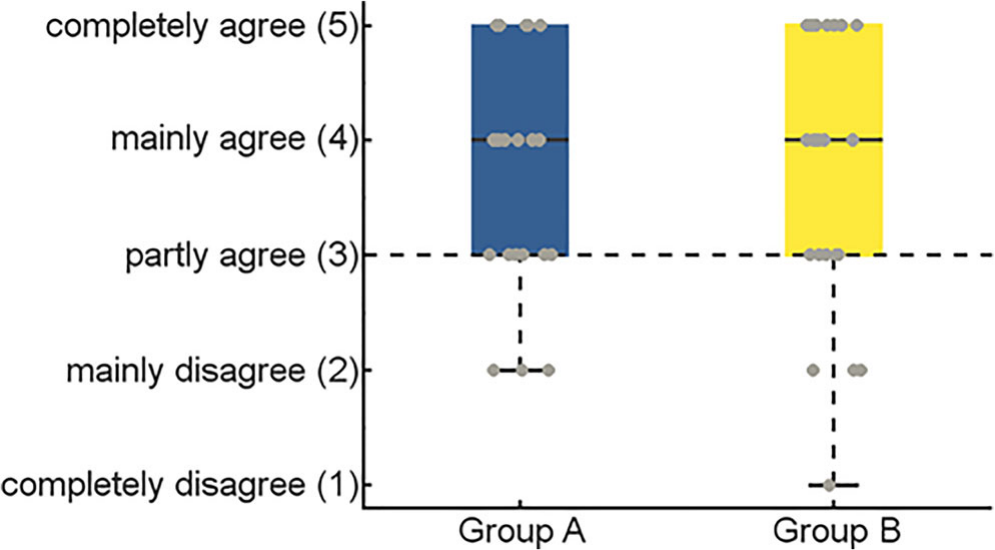
Boxplots showing the median score, interquartile range, and minimum and maximum of the data for Item 17 (“I had the impression to interact with a ‘real’ person.”), in blue for group A, in which the interaction partner was present, and in yellow for group B, in which the interaction partner was not present. The raw data are shown as gray dots.

Wilcoxon signed-rank test showed that the average score of Item 17 (*Mdn* = 4; *IQR* = 2.00; reflects the scale “mainly agree”), measuring whether the participants believed they were interacting with a real person, was significantly higher than the neutral score of 3 (*partly agree*), *W* = 680, *p* < .001, *r* = .591. A Mann–Whitney *U* test demonstrated that there was no significant difference in the experience of interacting with a real person between the two experimental groups (*Mdn*_*GA*_ = 4.00, *IQR* = 2.00), (*Mdn*_*GB*_ = 4.00, *IQR* = 2.00) as is depicted in Fig. 2, *U* = 302.5, *p* = .848, *r*_*rb*_ = .028. Seven out of 50 participants rated Item 17 below a score of 3; these included the two participants who scored a value of 1 in the plausibility criterion.

To analyze the experience of presence within the simulation, we calculated the median of all items for each concept (TP, CF, SRP, and CP). Table 2 lists the median and IQR of each item for the whole group and for groups A and B separately, as well as all statistics for the performed tests: A Wilcoxon signed-rank test for differences from the neutral score showed that all scores were significantly higher than the score of 3, indicating an increased experience of presence in the simulation. A Mann–Witney *U* test was performed for all four concept scores between the two groups, demonstrating that the experience of presence within the simulation did not differ between groups.

**Table 2:** Descriptive statistic of questionnaires for the experience of presence and statistical measures for testing group differences

					Wilcoxon Signed-Rank Test			Mann–Whitney *U* Test		
	Group	*N*	Median	IQR	*W*	*p*	*r*	*U*	*p*	Rank-Biserial Correlation
TP	WG	50	4.00	1.00	488.50	< .001	.564			
	A	25	4.00	1.00				300.00	.800	.038
	B	25	4.00	1.00						
CF	WG	50	3.00	1.00	369.00	.002	.432			
	A	25	3.00	1.00				292.50	.683	.060
	B	25	3.00	1.00						
SRP	WG	50	4.00	1.00	586.00	.001	.471			
	A	25	3.00	2.00				230.50	.097	.238
	B	25	4.00	1.25						
CP	WG	50	4.00	1.00	485.00	< .001	.496			
	A	25	4.00	1.00				297.50	.759	.045
	B	25	4.00	1.00						

WG, Whole group; A, group A; B, group B.

### Subjective experience of social agency

**Fig. 3:**
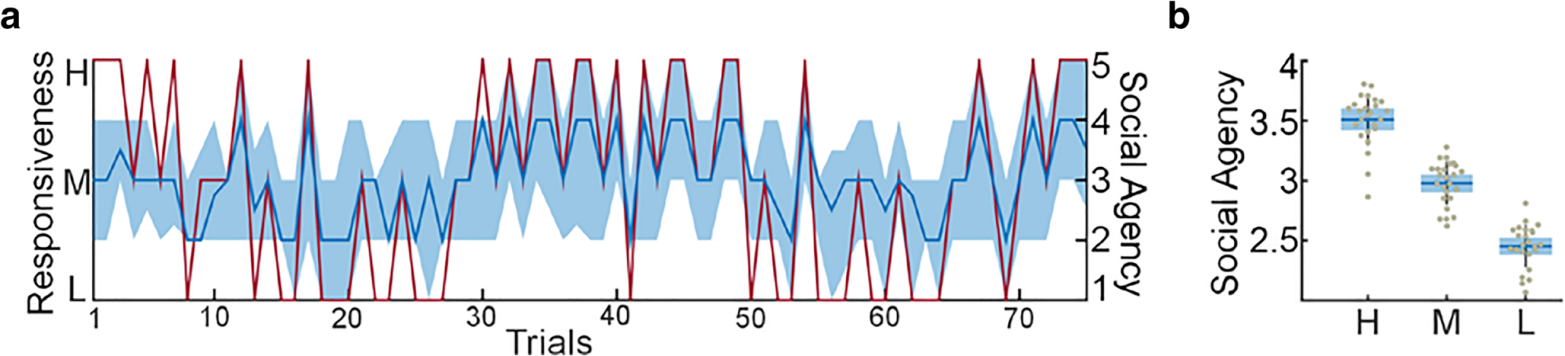
Subjective experience of social agency. (A) Time course of the trial-by-trial ratings. The simulated behavior of the interaction partner (experimental time course) is depicted as a red line; the group median is depicted as a blue line, whereas the lighter blue area indicates the interquartile range of the rated experience of social agency. (B) Averaged ratings for the different levels of responsiveness. Plots show the raw data points scattered over a 95% confidence interval (blue box) and one *SD* (vertical black line), as well as the mean (horizontal blue line).

To evaluate whether the levels of responsiveness modeled by simulating different gaze behaviors elicit different levels of explicit social agency, we calculated correlations between the time course of the median trial-by-trial score for the whole group and the time course of the experimentally controlled simulation. A Spearman’s rank correlation showed a strong association between the modeled level of responsiveness and rated social agency, *r*_*S*_ = .868, *p* < .001. Figure 3A depicts the time course of the modeled responsiveness of the simulation in red, and the median rating and IQR for each trial in blue. The mean ratings as well as the 95% confidence intervals and standard deviations (*SD*s) across participants for each level of responsiveness are depicted in Fig. 3B. A Mann–Whitney *U* test showed that the medians of the rated experience of agency were not significantly different between group A (*Mdn* = 3, IQR = 1; reflects a medium score) and group B (*Mdn* = 3, IQR = 1; reflects a medium score), *U* = 240, *p* = .980, *r*_*rb*_ = – .0056*.*

## Discussion

The present study presents three key findings: First, we demonstrated that our newly developed paradigm successfully simulates a believable gaze-based social interaction by using realistic video-stimuli. Furthermore, data suggests that the believability of this paradigm and the experience of presence within the simulation are not dependent on the actual presence of an interaction partner. Finally, the experience of social agency was shown to be dependent on the responsiveness of the simulation with respect to the contingency and congruency of the perceived gaze behavior. Therefore, the simulation can be considered useful for studying reciprocal feedback processed and the experience of social agency.

### Simulating social interactions

The validation of the simulation showed that few participants (4%) were aware of interacting with a pre-recorded video of an interaction partner instead of a real person. It therefore can be assumed that a core aspect of social interactions, namely the attribution of agency and mind to another person, was elicited by the simulation. Importantly, this was the case for both experimental groups, which indicates that the simulation is useful even without an additional experimenter present to create the cover story. Participants were mainly in accordance with the experienced concepts of presence (SP, TP, SRP, PC, and CF). It might be surprising that the median score for the different questionnaires was not higher and closer to the maximum score of 5, since most participants believed they were in a real interaction with another person. Due to the cover story, participants believed that the aim of the experiment was to evaluate digital communication. Therefore, participants might have compared the digital interaction in the paradigm to social interactions without a digital medium, which might have caused lower scores. Additionally, the reduced form of communication in the simulation, which was only based on gaze excluding emotional expressions and gestures, might have led to this result.

The two experimental groups did not differ in terms of their experiences of presence. This further emphasizes the possibility to simplify the experimental procedure and cover story for future studies with this type of paradigm. The experience of oneself and the interaction partner being present or involved in the simulation is specifically relevant for studying central aspects of human social behavior and also self-experiences. Previous research has shown that humans expect different behavior from computer-controlled than from human-controlled interaction partners (Pfeiffer et al., 2011; Wykowska et al., 2014). A functional magnetic resonance imaging study even showed different neural responses when people assumed they were interacting with a real person rather than with a computer (Pfeiffer et al., 2014). The expectations one has about the behavior of others are likely to be based on prior experiences gathered during development (Bowlby, 1977; Fonagy, Gergely, Jurist, & Target, 2004). People expect others to return eye-contact and follow their gaze in an interaction (Binetti, Harrison, Coutrot, Johnston, & Mareschal, 2016; Mareschal, Calder, & Clifford, 2013; Pfeiffer et al., 2011), particularly if it is not in a competitive context. For example, Pfeiffer et al. (2011) showed that gaze following is more likely to be expected and associated with a human- rather than a computer-controlled interaction partner. Such outcome expectations are likely to influence the sense of social agency since it is believed to strongly depend on the match between expected and perceived action outcome. The so-called *uncanny valley* is a related phenomenon found in interactions with artificial human characters (Mori, MacDorman, & Kageki, 2012). It describes the effect that viewing or interacting with nonhuman interaction partners that show a close to humanlike appearance elicit negative affect. A mismatch between the expected and perceived behavior is the assumed cause for this effect (Kätsyri, Förger, Mäkäräinen, & Takala, 2015). Simulated interaction partners that are either clearly nonhuman or that are believed to be actual interacting human beings will not lead to the reported negative experiences. The present experimental procedure therefore provides the possibility to study social interactions and particularly social agency in an experimentally controlled simulation without a bias caused by altered expectations or negative affect toward nonhuman interaction partners.

### Explicit social agency

In addition to the methodological advantages in studying social interaction, this study offers evidence that the responsiveness of an interaction partner influences the explicit sense of social agency within a continuous gaze-based social interaction. More precisely, gaze congruency (specifically gaze following and mutual gaze) and gaze contingency (reaction latency) seem to contribute toward these experiences. These findings are in line with previous noninteractive research using still pictures of faces, demonstrating a perceived sense of agency induced by gaze cues (Stephenson et al., 2018). In contrast to the implicit measure of agency employed in the study by Stephenson et al. (the intentional binding effect), the present experiment focuses on an explicit sense of social agency by asking participants to rate the intensity of the perceived relatedness of their own gaze towards the gaze of the interaction partner. Researchers in the field claim that the implicit and explicit sense of agency in motor actions might not relate to each other and are based on different processes (Dewey & Knoblich, 2014). It is unclear if this is also true within social interactions. Explicit ratings of experienced control together with measured implicit agency based on the temporal binding effects toward social gaze cues in the study of Stephenson et al. suggest these phenomena are related in a social context. According to the model of agency by Synofzik et al. (2013), the explicit judgment agency is more strongly related to cognitive aspects such as context, background beliefs and norms compared with implicit agency, which is described to be an automatic registration of being the cause of an action. A similar association can be assumed for the judgment of social agency. Furthermore, conscious explicit judgment and experience of being in control may also have a high impact on our feeling of responsibility, decision making and the resulting behavior (Frith, 2014; Haggard & Tsakiris, 2009; Karsh & Eitam, 2015; Moretto, Walsh, & Haggard, 2011). In terms of social interactions this suggests that one’s own experience of social agency might also alter how we react toward another person and in turn influence their experience of social agency. Social agency might therefore be an essential factor on interaction quality and the level of attachment between two people (Canevello & Crocker, 2010; Kleiman, Kashdan, Monfort, Machell, & Goodman, 2015).

In a clinical context, social agency could be an important aspect in the psychopathology of autism spectrum disorder (ASD) and could contribute to the understanding how individuals with ASD are perceived by persons without ASD. Since individuals with ASD show difficulties in sensing social cues particularly in online social interactions (Dratsch et al., 2013; Georgescu, Kuzmanovic, Roth, Bente, & Vogeley, 2014; Georgescu et al., 2013), it is likely that they have a decreased experience of social agency. Assuming that this also changes their behavior toward others it is not surprising that social interactions between individuals with ASD and without ASD are often described as being difficult. Individuals with ASD might in turn fail to elicit social agency in others and as a result are perceived as unresponsive and less engaged within an interaction. So far, the underlying mechanisms of such interaction difficulties have been attributed solely to the patient, but the concept of social agency is consistent with recent theoretical developments, which postulate that the disorders of social interaction depend not only on the patient, but rather on a mismatch between the perceived and expected reactions within the interaction (Bolis, Balsters, Wenderoth, Becchio, & Schilbach, 2017). Such discrepancies in social behavior are not only limited to ASD but also might occur in other mental health conditions with difficulties in processing social information. Future research on individuals across the whole autism spectrum would be needed to get a fuller understanding of how autistic traits influence the experience of social agency.

### Limitations

In the currently applied paradigm, the contingency and congruency of gaze responses are manipulated in a simplistic way: The contingency and congruency of the simulated behavior are modulated in parallel. The simulation is congruent completely, half of the time or not at all with correlated change in the contingency (response latencies: ~ 400 ms, ~ 700 ms, or not contingent) leading to three different stages of responsiveness. Therefore, it is not possible to disentangle the effects of each single factor to the reported social agency. Further research using this paradigm is needed to analyze how contingency and congruency of perceived gaze behavior interact to produce different experiences of responsiveness and social agency in participants. To achieve a fuller understanding of the mechanisms eliciting the experience of social agency, computational modeling might represent a useful tool for quantifying how the contingency and congruency of gaze behavior are integrated. In the present experiment, we assumed that the interactions and evaluations of social agency were not affected by the behavior during previous trials. However, it is possible that the behavior and evaluations of the participants could change on the basis of the first trials depending on the responsiveness of the interaction partner, which could be tested in future studies. Additionally, other important communicative aspects of social interactions such as the emotional expression are missing in the present version of the paradigm. The inclusion of dynamic facial stimuli showing different emotional expressions and analyzing the effects of this on social agency is another interesting research question that could be addressed with the paradigm in the future.

### Conclusion

Our results indicate that the novel experimental procedure presented here allows studying the experience of social agency in an ecologically valid and interactive manner even without the physical presence of a second person. This is of particular importance considering previous research showing how appearance and perceived agency of an interaction partner can influence one’s own experience within an interaction. Moreover, it was found that the sense of social agency was strongly dependent on the contingency and congruency of different gaze parameters such as gaze following, direct gaze and response latency. Further research is needed to evaluate how different social cues are integrated to form a combined sense of social agency within an interaction. Additionally, future studies should focus on the underlying neural networks and psychopathological mechanisms in patients with social interaction disorders that may be specifically characterized by alterations of the sense of social agency.

## Footnotes

1 Interrupted eye blinks or small-scale eye movements could conceivable reveal the transitions between video clips, due to sudden changes in eye or eye lid positions. However, when interacting with the agent, most gaze shifts of the agent happened in the peripheral vision of the participants, while they attended to one of the targets. Moreover, during pilot testing, our participants did not notice the video transitions, despite blinks. Thus, we did not need to apply video editing for blink suppression. However, recorded interaction partners showing excessive blink behavior were excluded from the stimulus materials.
